# A Digital Mental Health Intervention in an Orthopedic Setting for Patients With Symptoms of Depression and/or Anxiety: Feasibility Prospective Cohort Study

**DOI:** 10.2196/34889

**Published:** 2022-02-21

**Authors:** Ashwin J Leo, Matthew J Schuelke, Devyani M Hunt, John P Metzler, J Philip Miller, Patricia A Areán, Melissa A Armbrecht, Abby L Cheng

**Affiliations:** 1 Washington University in St Louis School of Medicine St Louis, MO United States; 2 Division of Biostatistics Washington University in St Louis School of Medicine St Louis, MO United States; 3 Division of Physical Medicine and Rehabilitation Department of Orthopedic Surgery Washington University in St Louis School of Medicine St Louis, MO United States; 4 Department of Psychiatry and Behavioral Sciences University of Washington Seattle, WA United States

**Keywords:** digital health, mental health, depression, anxiety, chronic pain, musculoskeletal, orthopedic, pain management, health intervention, mobile phone

## Abstract

**Background:**

Symptoms of depression and anxiety commonly coexist with chronic musculoskeletal pain, and when this occurs, standard orthopedic treatment is less effective. However, mental health intervention is not yet a routine part of standard orthopedic treatment, in part because of access-related barriers. Digital mental health intervention is a potential scalable resource that could be feasibly incorporated into orthopedic care.

**Objective:**

This study’s primary purpose was to assess the feasibility of introducing a digital mental health intervention (Wysa) in an outpatient orthopedic setting to patients with coexisting symptoms of depression and/or anxiety. The secondary purpose was to perform a preliminary effectiveness analysis of the intervention.

**Methods:**

In this single-arm, prospective cohort study, participants included adult patients (18 years and older) who presented to a nonsurgical orthopedic specialist at a single tertiary care academic center for evaluation of a musculoskeletal condition and who self-reported symptoms of depression and/or anxiety (Patient-Reported Outcomes Measurement Information System [PROMIS] Depression and/or Anxiety score ≥55). Face-to-face enrollment was performed by a research coordinator immediately after the participant’s encounter with an orthopedic clinician. Participants were provided 2 months of access to a mobile app called Wysa, which is an established, multicomponent digital mental health intervention that uses chatbot technology and text-based access to human counselors to deliver cognitive behavioral therapy, mindfulness training, and sleep tools, among other features. For this study, Wysa access also included novel, behavioral activation–based features specifically developed for users with chronic pain. Primary feasibility outcomes included the study recruitment rate, retention rate, and engagement rate with Wysa (defined as engagement with a therapeutic Wysa tool at least once during the study period). Secondary effectiveness outcomes were between-group differences in mean longitudinal PROMIS mental and physical health score changes at 2-month follow-up between high and low Wysa users, defined by a median split.

**Results:**

The recruitment rate was 29.3% (61/208), retention rate was 84% (51/61), and engagement rate was 72% (44/61). Compared to low users, high users reported greater improvement in PROMIS Anxiety scores (between-group difference −4.2 points, 95% CI −8.1 to −0.2; *P*=.04) at the 2-month follow-up. Between-group differences in PROMIS Depression (−3.2 points, 95% CI −7.5 to 1.2; *P*=.15) and Pain Interference scores (−2.3 points, 95% CI −6.3 to 1.7; *P*=.26) favored high users but did not meet statistical significance. Improvements in PROMIS Physical Function scores were comparable between groups.

**Conclusions:**

Delivery of a digital mental health intervention within the context of orthopedic care is feasible and has the potential to improve mental health and pain-related impairment to a clinically meaningful degree. Participants’ engagement rates exceeded industry standards, and additional opportunities to improve recruitment and retention were identified. Further pilot study followed by a definitive, randomized controlled trial is warranted.

**Trial Registration:**

ClinicalTrials.gov NCT04640090; https://clinicaltrials.gov/ct2/show/NCT04640090

## Introduction

### Background

Of the over 70 million Americans who have chronic musculoskeletal pain, up to half of them have a comorbid diagnosis of depression and/or anxiety [1-4]. Chronic pain and psychological impairment such as depression and anxiety are driven by shared biological pathways and neurotransmitters, and when a person has both conditions simultaneously, the effectiveness of standard treatment for either condition in isolation is reduced [5-7]. For example, among people who seek care for chronic musculoskeletal pain, those with coexisting depression and/or anxiety report worse pain, greater physical impairment, increased postoperative opioid use, and a lower rate of return to work than those without depression and anxiety [8-11].

Despite strong evidence regarding the interconnection between physical pain and mental health, psychological assessment and intervention is not yet considered a routine part of orthopedic treatment. This is, in part, because of both provider- and patient-related barriers. Orthopedic clinicians do not feel equipped with the necessary time and referral resources to address mental health [12], and patients report barriers to seeking mental health treatment even outside an orthopedic setting. Among people who recognize their need for mental health treatment, 64% are resistant to reaching out to another person for assistance and 23% have financial, transportation, or time-related barriers to seeking care [13,14]. Even if all patients had the resources to seek appropriate care, unmet need would persist because of a national shortage of qualified mental health clinicians [15].

Digital mental health interventions address the accessibility-, cost-, convenience-, and stigma-related barriers to traditional in-person mental health treatment [16]. Furthermore, a growing body of evidence supports the effectiveness of digital mental health interventions in improving depression and anxiety symptoms, sometimes to a degree equivalent to that obtained from in-person treatment [17-19]. With the widespread, everyday use of smartphones, these emerging digital tools could function as efficient, widely available mental health resources for orthopedic clinicians to offer their patients [16,20-22].

### Objectives

The primary purpose of this study was to assess the feasibility of introducing a digital mental health intervention (Wysa) in an outpatient orthopedic setting to patients with musculoskeletal pain and coexisting symptoms of depression and/or anxiety. The secondary purpose was to perform a preliminary effectiveness analysis of the intervention. We hypothesized that delivery of a digital mental health intervention would be feasible in an orthopedic clinic setting. Furthermore, compared to “low users” of the digital mental health intervention, “high users” would report greater improvements in mental and physical health symptoms at the 2-month follow-up.

## Methods

### Design

This was a single-arm, prospective cohort, pilot feasibility study performed at a tertiary care academic medical center in the United States. Institutional review board approval was obtained prior to participant recruitment (IRB #202005219), and the study was registered through ClinicalTrials.gov (NCT04640090). Participants were enrolled from December 8, 2020, through July 14, 2021, with some interruptions related to the COVID-19 pandemic.

### Participants

#### Eligibility Criteria

Participants were recruited from among patients who presented to an orthopedic department for evaluation and treatment of a musculoskeletal condition. To be eligible, patients had to be adults (18 years or older) presenting to a nonsurgical orthopedic provider. Additionally, patients had to have symptoms of depression and/or anxiety as indicated by a score of 55 or higher on the Patient-Reported Outcomes Measurement Information System (PROMIS) Depression and Anxiety measures; this is collected as standard clinical care on check-in for each outpatient appointment at this orthopedic department. Patients who were actively planning to start in-person mental health treatment were excluded from participation. Patients without access to a mobile device were also not eligible.

#### Recruitment Process

We recruited participants from among patients who presented to 6 orthopedic clinicians, 5 of whom were board-certified physical medicine and rehabilitation physicians (physiatrists) with subspecialty training in spine and sports medicine and 1 who was a nurse practitioner with 11 years of nonsurgical orthopedics experience. Prior to the clinician’s encounter with each patient, the study coordinator notified the clinician of patients who met the age and PROMIS score criteria for the study. Then, at the end of a potentially eligible patient’s standard clinic encounter, the clinician briefly introduced the study. For those who expressed initial interest, the research coordinator approached the patient, provided further study details, and completed the final screening, consent, and enrollment process for those who were interested. When possible, participant enrollment was completed in person before the participant left the orthopedic clinic office. Patients who were interested in the study but did not have time to enroll immediately were contacted over the phone by the research coordinator to complete the enrollment process. No compensation besides access to the study intervention was provided to participants for their participation in the study.

### Intervention

**Figure 1:**
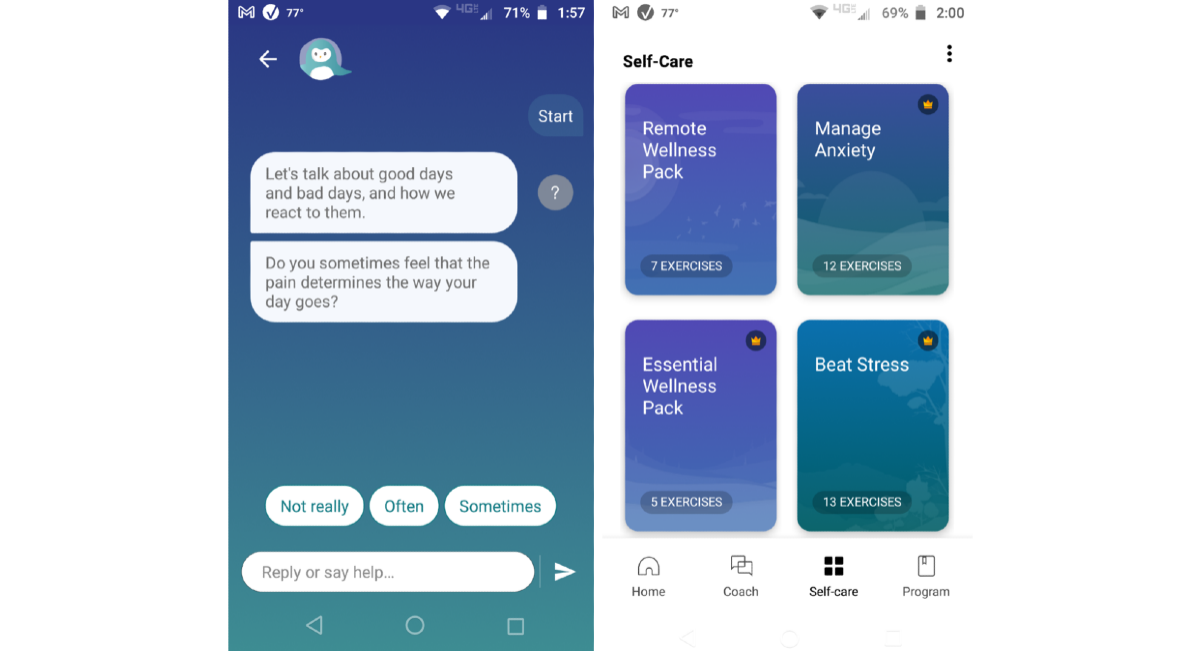
Screenshots of Wysa’s chronic pain version.

All study participants received a complimentary, 2-month subscription to Wysa, which is a commercially available digital mental health intervention. Wysa is a multicomponent intervention that uses artificial intelligence–based chatbot technology and human “coaches” (counselors) with master’s degrees in psychology to deliver therapeutic content such as cognitive behavioral therapy, dialectical behavioral therapy, motivational interviewing, mindfulness training, deep breathing techniques, and sleep meditations [23,24]. Commercially, basic chatbot features are freely available, and premium digital features and text-based sessions with a human coach are available for a monthly fee. In this study, participants received access to a novel version of Wysa that was specifically developed for users interested in tools to manage chronic pain (Figure 1). Using behavioral activation and pain acceptance principles, this pain-specific version of Wysa encourages users to “engage with things that bring joy, despite having pain” [25-28]. Added features include daily check-ins, weekly reports, the ability to unlock premium “reward” tool packs by engaging with the weekly reports, and a progress roadmap. The chatbot and human coach features are also still embedded within the pain version of Wysa. This version is not yet part of the Wysa commercial product.

### Variables

Data were obtained from 3 sources: electronic medical records, participants’ self-report, and usage reports from Wysa. Data collected as standard clinical care were obtained from the electronic medical record; this included basic sociodemographic variables, medical history, and pain medication use. Additionally, on the day of the orthopedic clinic visit and study enrollment, patients completed Adult PROMIS CAT Depression v1.0, Anxiety v1.0, Pain Interference v1.1, and Physical Function v2.0 measures as standard care prior to meeting with the clinician. These measures were completed on a tablet computer (iPad Mini; Apple Inc). Each PROMIS domain is normalized to the general United States population with a mean (SD) score of 50 (SD 10) [29]. Higher scores represent more of the domain; for instance, a score of 60 on PROMIS Anxiety indicates anxiety symptoms that are worse than those in the general population, but a score of 60 on PROMIS Physical Function indicates physical function that is better than that in the general population.

On study enrollment, participants completed additional self-reported measures on the tablet computers. These included participants’ pain duration, primary pain location, height and weight (to calculate BMI), history of tobacco use, exercise habits, Brief Resilience Scale [30], and Importance, Readiness, and Confidence to Change rulers [31].

At the 1- and 2-month follow-up, participants were emailed a questionnaire that included PROMIS Depression, Anxiety, Pain Interference, and Physical Function domains; participants received automated email reminders as needed 2 and 4 days later. For the 2-month follow-up, they also received up to 3 phone call reminders if they had not completed the questionnaire by the sixth day after the follow-up period. All electronic medical record and self-reported data were stored in a secure electronic Research Electronic Data Capture (REDCap; Vanderbilt University) database [32,33].

Wysa provided user-specific, timestamped usage data for each app interaction by participants. Individual users were identified with randomly generated codes, and only study personnel had access to the participant key. No personally identifiable data were shared with Wysa by the study team or vice versa.

### Outcomes

#### Feasibility

The primary study outcomes were related to the feasibility of introducing a digital mental health intervention (Wysa) in an outpatient orthopedic setting and conducting a fully powered, randomized controlled effectiveness trial in this setting. Outcomes included the study recruitment rate (defined as the number of patients who signed the study informed consent document divided by the number of patients approached for participation), study retention rate (defined as the proportion of recruited participants who completed the 2-month follow-up PROMIS measures), and the rate of engagement with Wysa (defined as the proportion of recruited participants who interacted with Wysa at least once after onboarding to the app). Other engagement metrics were also evaluated, including the weekly engagement rate (defined as the proportion of recruited participants who completed at least 8 interactions with Wysa over the 8-week period, because this would approximately mirror the intensity of weekly sessions with an in-person therapist), the coach engagement rate (defined as the proportion of recruited participants who completed at least one session with a human coach), and the most frequently used features within Wysa identified in a descriptive analysis. An exploratory analysis was also conducted to assess for differences in patient demographics (ie, age, sex) between those who enrolled in this study and those who did not.

#### Effectiveness

A secondary purpose of the study was to perform a preliminary effectiveness analysis of the intervention. To account for clinical improvements that could be related to usual orthopedic care or natural variations in symptom severity over time, the effectiveness outcomes considered were the between-group differences (between high and low Wysa users) in 2-month changes on PROMIS Depression, Anxiety, Pain Interference, and Physical Function measures. High versus low Wysa usage was defined by a participant’s number of total Wysa interactions lying above versus below the median engagement level among participants. Minimum clinically meaningful effect sizes were a priori chosen in accordance with previously published literature involving patients with chronic musculoskeletal pain who were managed nonoperatively. Effect sizes also had to exceed the SE of measurement for each PROMIS computer adaptive test at the study institution to be considered meaningful. Therefore, clinically meaningful effect sizes were defined as at least 3.2 points for PROMIS Depression, 3.0 points for Anxiety, 2.0 points for Pain Interference, and 2.2 points for Physical Function [34-36].

### Statistical Analysis

Univariate descriptive statistics were calculated for all study variables. Age and sex were compared between those who did and did not enroll using an unequal-variances *t* test and a two-sample chi-square test of proportions, respectively. A median split on the total number of Wysa interactions was chosen to categorize participants as high and low Wysa users because density plots of the distributions of Wysa total interactions, chatbot interactions, and coach sessions failed to identify any empirical cut points. For the effectiveness analyses, linear mixed-effects models were fit predicting each of the 4 PROMIS measures from Wysa usage status (high versus low) and month. Adjusting for age as a covariate in the models did not meaningfully influence the model results, so unadjusted models are reported. Missing data were omitted. Significance was set a priori at *P*<.05, and sample size for this feasibility study was determined by the availability of resources. Statistical analyses were performed using R (v4.0.2; R Core Team).

## Results

### Feasibility

**Figure 2:**
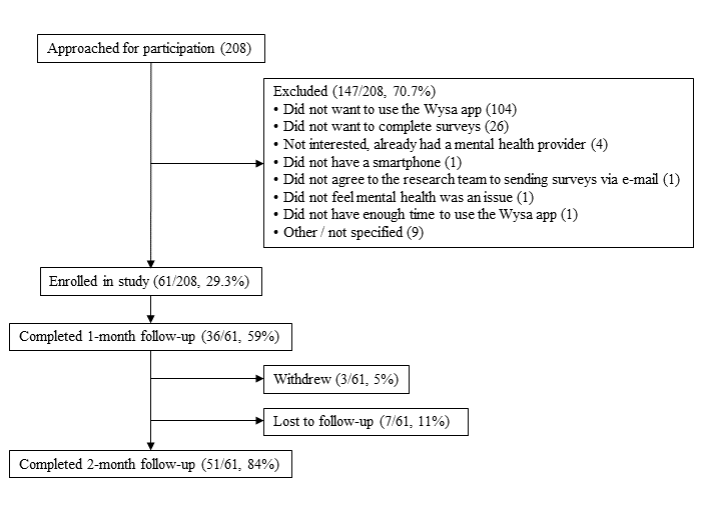
Participant flow, from initial study contact through final follow-up.

The study recruitment rate was 29.3% (61/208; Figure 2). Among the 61 patients who enrolled in the study, the median age was 55 years (range 18-83 years) and 87% (53/61) were female (Tables 1 and 2). The most common reasons patients did not enroll were that they did not want to use the app (104/147, 70.7%) or did not want to complete the research surveys (26/147, 17.7%). No statistically significant differences were observed in the age or sex distribution of patients who enrolled versus those who did not enroll in the study (*P*=.21; Table 3).

The retention rate of participants who consented to participate in the study was 84% (51/61). More participants completed the 2-month follow-up measures (51/61, 84% using email and phone reminders) than the 1-month follow-up measures (36/61, 59% using email reminders only).

The overall rate of engagement with Wysa among participants who consented to participate in the study was 72% (44/61). The weekly engagement rate was 57% (35/61), and the coach engagement rate was 33% (20/61). The therapeutic mechanisms underlying the tools most commonly used by participants were related to mindfulness (122/351, 34.8%), cognitive behavioral therapy (76/351, 21.7%), and sleep (53/351, 15.1%; Multimedia Appendix 1). Of the rewards unlocked by participants, sleep-related rewards were the most common (16/48, 33%) Table 4).

**Table 1:** Baseline characteristics of patients who presented to an orthopedic clinic for a musculoskeletal condition, reported coexisting symptoms of depression and/or anxiety, and enrolled in a research study that provided access to a digital mental health intervention (Wysa) (N=61).

Characteristic		Value
Age (years), median (IQR)		55 (42-64)
**Sex, n (%)**		
	Female	53 (87)
	Male	7 (12)
	Transgender	1 (2)
**Race, n (%)**		
	White/Caucasian	55 (90)
	Black/African American	6 (10)
**Ethnicity, n (%)**		
	Hispanic	2 (3)
	Not Hispanic	59 (97)
**Area Deprivation Index^a^, n (%)**		
	Quartile 1 (least deprived)	19 (31)
	Quartile 2	15 (25)
	Quartile 3	14 (23)
	Quartile 4 (most deprived)	13 (21)
Pain duration (years), median (IQR)		2 (0.9-5.9)
**Pain location^b^**		
	Low back	31 (51)
	Leg	19 (31)
	Neck	18 (30)
	Arm	8 (13)
	Generalized pain	3 (5)
BMI (kg/m^2^), mean (SD)		29 (7)
Tobacco use, n (%)		4 (7)
Moderate/strenuous exercise (minutes/week), median (IQR)		40 (0-120)
Exercise limited by pain, n (%)		49 (85)
Exercise limited by enjoyment, n (%)		6 (11)
Brief Resilience Scale score^c^, median (IQR)		3.2 (2.5-3.7)
**Potential for behavior change^d^, median (IQR)**		
	Importance of change	76 (68-90)
	Readiness for change	69 (50-82)
	Confidence in the ability to change	68 (50-74)
Any pain medication use, n (%)		46 (75)
**Specific pain medication use^e^, n (%)**		
	Opioid	7 (15)
	Nonsteroidal anti-inflammatory drug	15 (33)
	Neuropathic	22 (48)
	Other	20 (44)
**Medical history, n (%)**		
	Hypertension	18 (30)
	Hyperlipidemia	17 (28)
	Cardiovascular disease	7 (11)
	Diabetes	8 (13)
	Sleep apnea	9 (15)
	Depression	24 (39)
	Anxiety	23 (38)

^a^The national Area Deprivation Index is a community-level measure of social disadvantage based on a person’s 9-digit zip code [37,38].

^b^Some patients reported multiple pain locations.

^c^The Brief Resilience Scale is scored from 1 to 5, with higher scores representing greater resilience [30].

^d^Potential for change measures are scored from 0 to 100, and higher scores are favorable [39].

^e^Number of patients among the 46 patients who used any pain medication.

**Table 2:** Summary of mental and physical health scores measured by the Patient-Reported Outcomes Measurement Information System over the 2-month follow-up in patients who presented to an orthopedic clinic for a musculoskeletal condition, reported coexisting symptoms of depression and/or anxiety, and enrolled in a research study that provided access to a digital mental health intervention (Wysa).

PROMIS^a^ scores	Baseline (n=61), mean (SD)	2-month follow-up (n=51), mean (SD)
Depression	58.1 (7.3)	54.7 (8.7)
Anxiety	62.2 (5.9)	58.0 (7.8)
Pain interference	65.2 (6.5)	62.1 (7)
Physical function	35.9 (6.6)	39.5 (6.7)

^a^PROMIS: Patient-Reported Outcomes Measurement Information System.

**Table 3:** Demographic comparison of patients who presented to an orthopedic clinic for a musculoskeletal condition, reported coexisting symptoms of depression and/or anxiety, and enrolled versus those who did not enroll in a research study that provided access to a digital mental health intervention (Wysa) (N=208).

Demographic	Enrolled (n=61)	Did not enroll (n=147)	Between-group difference	
			Mean difference or % difference (95% CI)	*P* value
Age (years), mean (SD)	53 (14)	56 (18)	3 (−2 to 8)	.21
Female sex, n (%)	53 (87)	115 (78.2)	9 (−3 to 21)	.21

**Table 4:** Summary of engagement with a digital mental health intervention (Wysa) by patients who presented to an orthopedic clinic for a musculoskeletal condition and reported coexisting symptoms of depression and/or anxiety (N=61).

Engagement metric	Median (IQR)	Range
Total interactions	18 (0-64)	0-544
Interactions with chatbot	9 (0-29)	0-181
Daily check-ins completed	2 (0-16)	0-81
Weekly reports viewed	0 (0-2)	0-14
Rewards unlocked	0 (0)	0-8
Text-based sessions with a human coach	0 (0-1)	0-12
Total messages to a human coach	0 (0-27)	0-382

### Effectiveness

**Figure 3:**
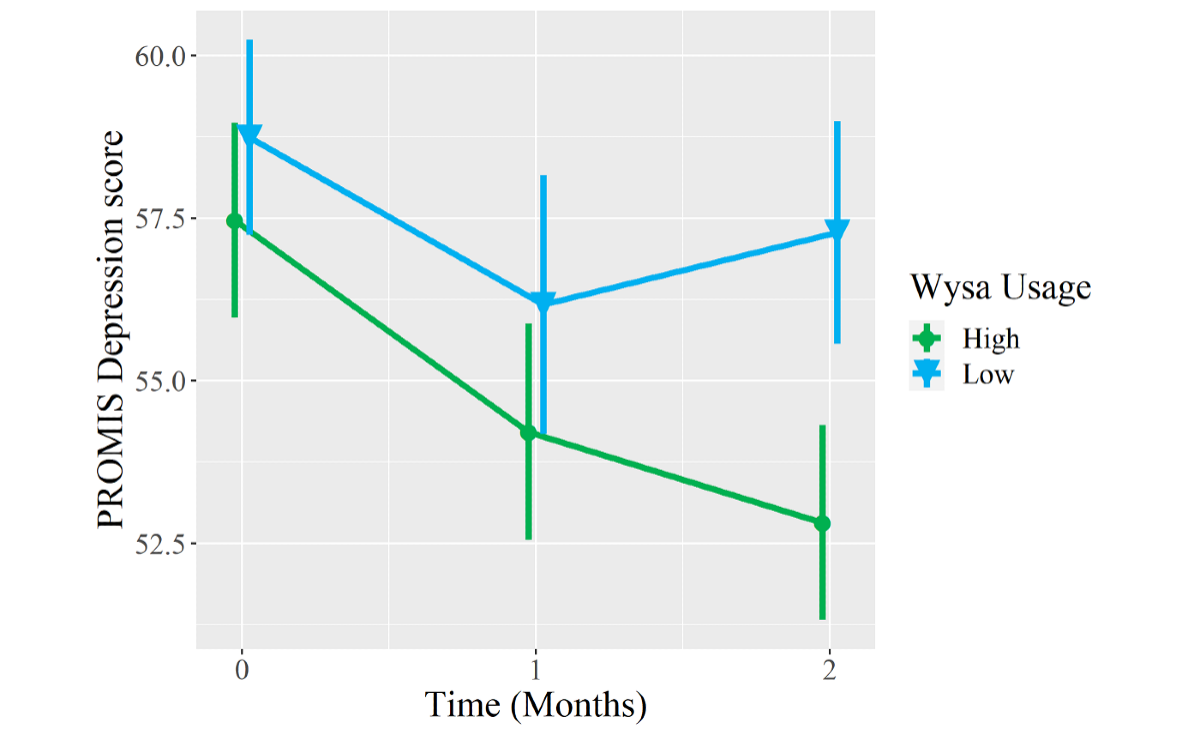
Mean longitudinal change in Patient-Reported Outcomes Measurement Information System (PROMIS) Depression scores over 2-month follow-up in patients who presented to an orthopedic clinic for a musculoskeletal condition, reported coexisting symptoms of depression and/or anxiety, and were high users (n=30, green circles) versus low users (n=31, blue triangles) of a digital mental health intervention (Wysa). Error bars represent standard errors.

**Figure 4:**
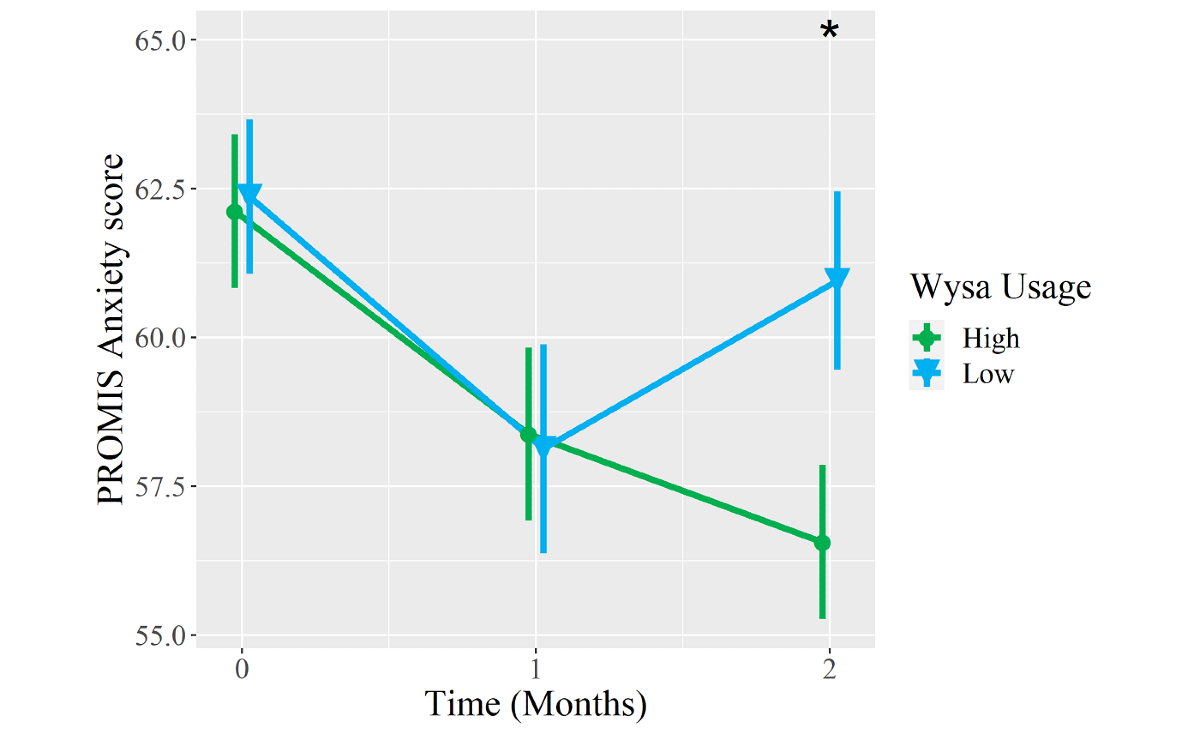
Mean longitudinal change in Patient-Reported Outcomes Measurement Information System (PROMIS) Anxiety scores over 2-month follow-up in patients who presented to an orthopedic clinic for a musculoskeletal condition, reported coexisting symptoms of depression and/or anxiety, and were high users (n=30, green circles) versus low users (n=31, blue triangles) of a digital mental health intervention (Wysa). Error bars represent standard errors. Asterisk represents a statistically significant between-group difference in longitudinal score changes from baseline to 2-month follow-up (*P*<.05).

**Figure 5:**
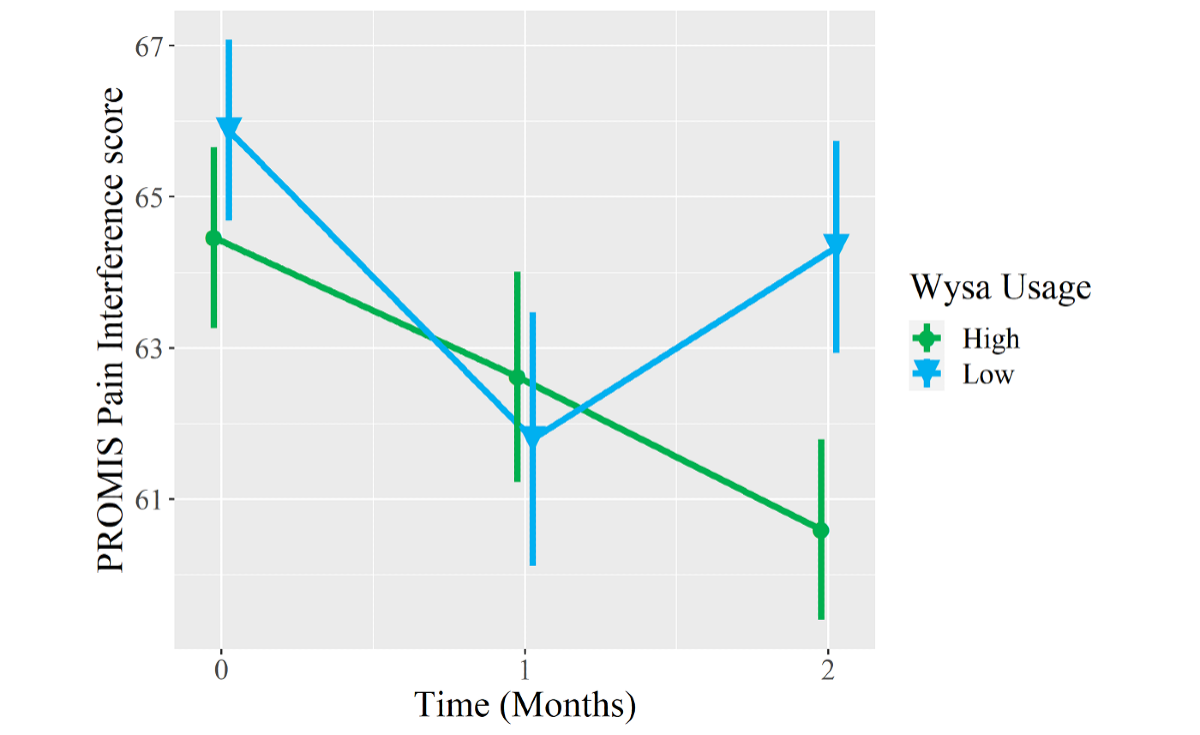
Mean longitudinal change in Patient-Reported Outcomes Measurement Information System (PROMIS) Pain Interference scores over 2-month follow-up in patients who presented to an orthopedic clinic for a musculoskeletal condition, reported coexisting symptoms of depression and/or anxiety, and were high users (n=30, green circles) versus low users (n=31, blue triangles) of a digital mental health intervention (Wysa). Error bars represent standard errors.

**Figure 6:**
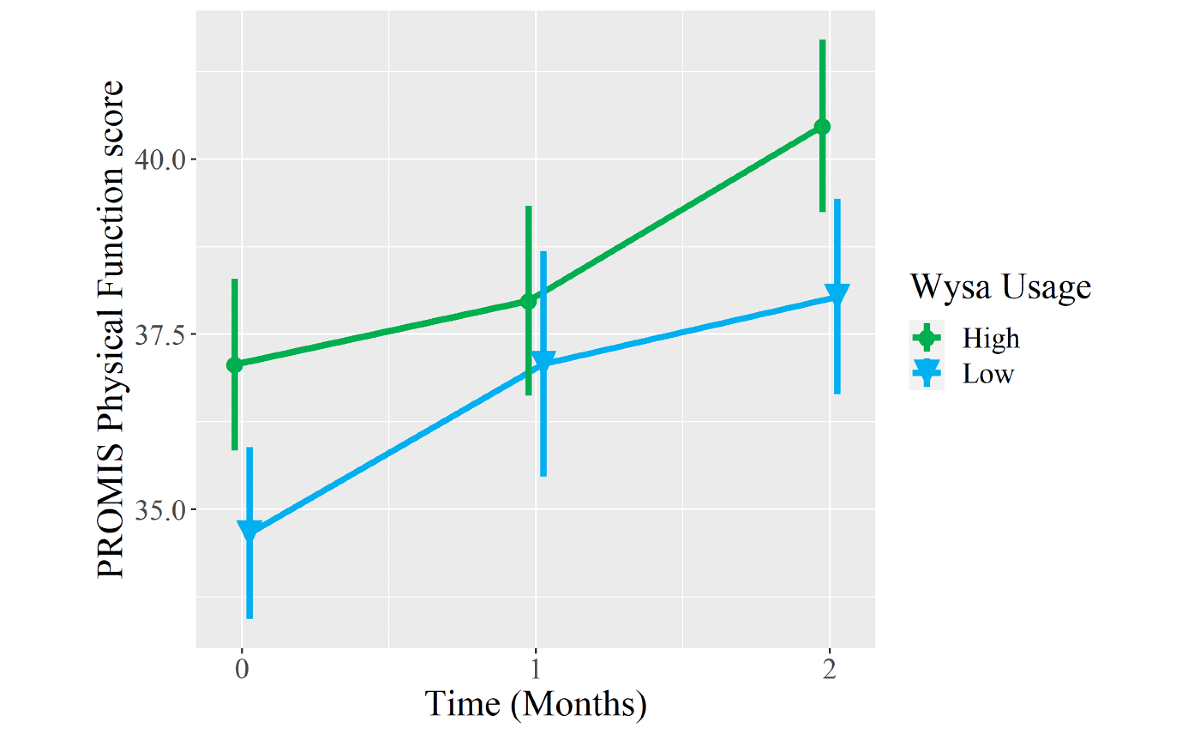
Mean longitudinal change in Patient-Reported Outcomes Measurement Information System (PROMIS) Physical Function scores over 2-month follow-up in patients who presented to an orthopedic clinic for a musculoskeletal condition, reported coexisting symptoms of depression and/or anxiety, and were high users (n=30, green circles) versus low users (n=31, blue triangles) of a digital mental health intervention (Wysa). Error bars represent standard errors..

Compared to low Wysa users, high Wysa users reported greater improvement to a clinically meaningful degree in PROMIS Anxiety scores at the 2-month follow-up (between-group difference −4.2 points, 95% CI −8.1 to −0.2; *P*=.04; Table 5). Between-group differences in 2-month improvements in PROMIS Depression (−3.2 points, 95% CI −7.5 to 1.2; *P*=.15) and Pain Interference scores (−2.3 points, 95% CI −6.3 to 1.7; *P*=.26) favored high Wysa users and met clinically meaningful thresholds but did not meet statistical significance. In contrast, both groups reported clinically meaningful and statistically significant improvements in PROMIS Physical Function scores from baseline to 2 months (*P*=.99), but there was no between-group difference in this domain (Figures 3-6).

**Table 5:** Summary of mental and physical health changes (measured by the Patient-Reported Outcomes Measurement Information System) across the 2-month follow-up in patients who presented to an orthopedic clinic for a musculoskeletal condition and reported coexisting symptoms of depression and anxiety subgrouped by frequency of use of a digital mental health intervention (Wysa).

PROMIS^a^ domain		Baseline (n=61), mean (95% CI)	2-month follow-up (n=51)^b^, mean (95% CI)	Within-group longitudinal change, mean (95% CI)	Between-group change^c^	
					Mean (95% CI)	*P* value
**Depression**					−3.2 (−7.5 to 1.2)	.15
	High users^d^	57.5 (54.5 to 60.4)	52.8 (49.9 to 55.8)	−4.7 (−7.5 to −1.8)		
	Low users^e^	58.7 (55.8 to 61.7)	57.3 (53.9 to 60.7)	−1.5 (−4.7 to 1.8)		
**Anxiety**					−4.2 (−8.1 to −0.2)	.04
	High users	62.1 (59.6 to 64.7)	56.6 (54.0 to 59.1)	−5.6 (−8.2 to −3.0)		
	Low users	62.4 (59.8 to 64.9)	61.0 (58.0 to 63.9)	−1.4 (−4.4 to 1.6)		
**Pain interference**					−2.3 (−6.3 to 1.7)	.26
	High users	64.5 (62.1 to 66.8)	60.6 (58.2 to 63.0)	−3.9 (−6.5 to −1.2)		
	Low users	65.9 (63.5 to 68.3)	64.3 (61.6 to 67.1)	−1.5 (−4.6 to 1.5)		
**Physical function**					0.0 (−3.4 to 3.5)	.99
	High users	37.1 (34.6 to 39.5)	40.5 (38.0 to 42.9)	3.4 (1.2 to 5.7)		
	Low users	34.7 (32.3 to 37.1)	38.0 (35.3 to 40.8)	3.4 (0.8 to 6.0)		

^a^PROMIS: Patient-Reported Outcomes Measurement Information System.

^b^The 2-month follow-up PROMIS measures were available for 30 high and 21 low Wysa users.

^c^Clinically meaningful effect sizes are defined as at least 3.2 points for PROMIS Depression, 3.0 points for Anxiety, 2.0 points for Pain Interference, and 2.2 points for Physical Function [34-36].

^d^There were 30 patients in the high Wysa use subgroup.

^e^There were 31 patients in the low Wysa use subgroup.

## Discussion

### Principal Results

Intervention for mental health, which is a comorbidity that so often accompanies musculoskeletal pain, can improve both mental health- and pain-related impairment in patients who present to an orthopedic clinic for a musculoskeletal condition. To address mental health in the orthopedic setting, an acceptable, scalable, effective intervention must be available. In this pilot study, we investigated the feasibility and potential for effectiveness of delivering the digital mental health intervention Wysa in an orthopedic setting to patients with coexisting symptoms of depression and/or anxiety. The recruitment rate was 29.3% (61/208) across 6 orthopedic providers and the retention rate was 84% (51/61) with respect to completion of the 2-month outcome measures. Of the enrolled participants, 72% (44/61) engaged with Wysa at least once during the study period. Compared to low Wysa users, high Wysa users reported greater improvements by 4.2 points in PROMIS Anxiety scores at the 2-month follow-up. This difference is statistically significant (*P*=.044) and meets the predetermined threshold for clinical meaningfulness. Between-group differences in 2-month improvement also favored high Wysa users for PROMIS Depression (by 3.2 points) and PROMIS Pain Interference (by 2.3 points); these differences met clinically meaningful thresholds, but they did not meet statistical significance in this pilot feasibility study.

### Implications

These findings suggest that a fully powered, randomized controlled trial is feasible and warranted to assess the clinical benefit of offering a digital mental health intervention in the context of orthopedic care for patients with coexisting symptoms of depression and/or anxiety. Engagement in our study was notably higher than digital health industry standards, which measure engagement on the order of days instead of weeks or months [40]. The high engagement rates and positive clinical findings in this study are consistent with those in a study of 10,000 participants by Bailey et al [41], in which people with chronic low back and/or knee pain were given a 12-week digital health intervention sponsored by their employers [41]. In that study, 73% of participants continued engagement with the program into the third month. We hypothesize that introducing a digital health intervention through a trusted resource such as a physician or employer may contribute to these higher engagement rates. Furthermore, if an orthopedic provider referred a patient to a digital mental health intervention as part of standard care (as opposed to a research study), we hypothesize that rates of onboarding to the intervention would be even greater than the enrollment rates we observed in this study. Patients who present to an orthopedic clinic for a musculoskeletal condition have demonstrated a willingness to engage with digital (physical) health interventions [42], and incorporation of digital mental health support is a logical next step to improving clinical outcomes in orthopedic care.

This line of study may also have implications for ambulatory care settings other than orthopedic clinics. We believe that clinical encounters related to chronic pain are a particularly opportune setting to address mental health because when patients understand that their physical pain experience is directly linked to their mental health, their desire to reduce pain becomes a tangible (and potentially nonstigmatizing) motivator for addressing mental health. This approach of inserting a mental health access point and resources could be applied to other frequently used ambulatory care settings that address chronic pain (eg, primary care, rheumatology, gastroenterology, headache clinics) [43], and it would create opportunities to provide mental health screening and intervention for patients with symptoms of depression and/or anxiety who might not have otherwise sought mental health treatment. In our experience, the orthopedic setting poses some especially challenging barriers to the discussion of mental health with patients, which include patients’ expectations regarding appropriate content to discuss during the clinic encounter and providers’ lack of training and possible lack of inherent interest or budgeted time to discuss mental health with patients. Therefore, evidence of the feasibility of delivering mental health intervention within the orthopedic setting is certainly encouraging for potential success in other ambulatory care settings that address chronic pain. Furthermore, although no coordination was made with patients’ established mental health care providers in this study (for those who were already receiving some form of mental health treatment), better care coordination and communication between nontraditional mental health “access points” (eg, within orthopedics) and patients’ established mental health providers should be focused on in the future.

### Lessons Learned

During this feasibility study, we gained insight into participant recruitment and retention that we anticipate can be used to further improve these rates in a subsequent fully powered randomized controlled trial. Regarding participant recruitment, although every orthopedic provider that we approached was willing to assist in recruitment, it was clear that their comfort in initiating a conversation about mental health with patients varied widely. Providers who were initially less comfortable were receptive to brief training with short scripts that could be used during patient-provider discussions, and their comfort level subjectively improved through the recruitment period. The research coordinator’s conversation with patients also evolved during the study as she learned the language and content that were most acceptable and compelling to patients (ie, “this app is designed to help with stress and well-being” as opposed to “this is a mental health app”). We are pursuing further pilot work to obtain a more comprehensive understanding of (surgical and nonsurgical) orthopedic provider and patients’ needs regarding discussion of mental health in an orthopedic setting, both in a clinical and research context. This additional work will be essential to facilitate widespread patient engagement with a mental health intervention that is introduced in an orthopedic context.

Regarding participant retention, integration of phone call reminders improved the follow-up survey response rate compared to the use of email reminders alone. However, many participants were still hesitant to answer phone calls and voicemails were left when possible. For future trials, we plan to integrate SMS text messaging–based reminders as well. We will also compensate participants not for use of the intervention but for completion of each follow-up survey. We anticipate these protocol changes to further improve our recruitment and retention rates in subsequent trials.

### Strengths and Limitations

The primary strength of this feasibility study is that to our knowledge, it is the first investigation to incorporate a digital mental health intervention into an orthopedic care setting. The primary limitation is that there was no true control arm because of a lack of available resources. To account for longitudinal improvements that may have resulted from the usual orthopedic care that patients received during the study period (eg, physical therapy, injections, pain medications), we compared high versus low users of Wysa in our preliminary effectiveness analysis. However, healthy participant bias could still have been present; that is, high Wysa users may have been more ready than low Wysa users to address their mental health, and the same phenomenon could have occurred between patients who chose to participate and those who did not participate in the study. Nevertheless, successful completion of this feasibility study suggests that at least some orthopedic patients are interested in receiving mental health intervention as a component of their orthopedic care.

### Generalizability

While the study findings offer insight into important next steps for this research, the generalizability of the results of this current single-center study is somewhat limited. Collection of mental health screening measures is becoming more commonplace in orthopedic clinics, but it is still not performed universally [44], and collection of these measures may have facilitated the recruitment process for our study. Comfort level and success in discussing mental health in an orthopedic setting are also likely provider-dependent [12]. The recruitment of participants from among patients who presented to 6 providers at our institution improves the generalizability of our findings, but all providers were nonsurgical specialists. Furthermore, we observed variations among providers with respect to the introduction of the study to the patients and the longitudinal refinement of each provider’s approach during the study. From an implementation standpoint, further work is also needed to understand how a digital mental health intervention could most effectively and efficiently be delivered to patients who present to an orthopedic clinic for a musculoskeletal condition when a research coordinator is not available to assist the patient with onboarding to the app. We are actively investigating these provider-level and implementation-related limitations to generalizability in further pilot work.

### Conclusions

In summary, this pilot study explored the feasibility and potential for effectiveness of delivering the digital mental health intervention Wysa in an orthopedic setting to patients with coexisting symptoms of depression and/or anxiety. We demonstrated that it is possible for orthopedic providers to introduce a mental health intervention to these patients. Furthermore, high users of the intervention reported greater improvement in anxiety symptoms than low users at the 2-month follow-up. High users of the intervention may also achieve meaningfully greater 2-month improvements in depression and pain interference, but this study was not powered to detect these differences. It is valuable to continue this line of research because it offers potential to improve both mental health and pain-related impairment in people with chronic musculoskeletal pain and coexisting depression and/or anxiety in a manner that is feasible and scalable to deliver in an orthopedic setting. More pilot work is needed (and is ongoing) to optimize the ability of orthopedic providers to efficiently discuss mental health with these patients in a manner that is mutually acceptable. Ultimately, a fully powered randomized controlled trial is warranted, and this approach of inserting mental health access points and resources into non–mental health ambulatory care settings could also be considered in primary care and other specialty clinics that often address chronic pain.

## Appendix

Multimedia Appendix 1 Distribution of therapeutic mechanisms underlying the Wysa tools used by 61 patients who presented to an orthopedic clinic for a musculoskeletal condition and coexisting symptoms of depression and anxiety. CBT: cognitive behavioral therapy.

## Abbreviations

PROMIS: Patient-Reported Outcomes Measurement Information System
REDCap: Research Electronic Data Capture
