# Supplementary figures and images for: A Digital Mental Health Intervention in an Orthopedic Setting for Patients With Symptoms of Depression and/or Anxiety: Feasibility Prospective Cohort Study
Source: JMIR Form Res. 2022 Feb 21;6(2):e34889. doi: 10.2196/34889 (PMC8902664; doi:10.2196/34889)

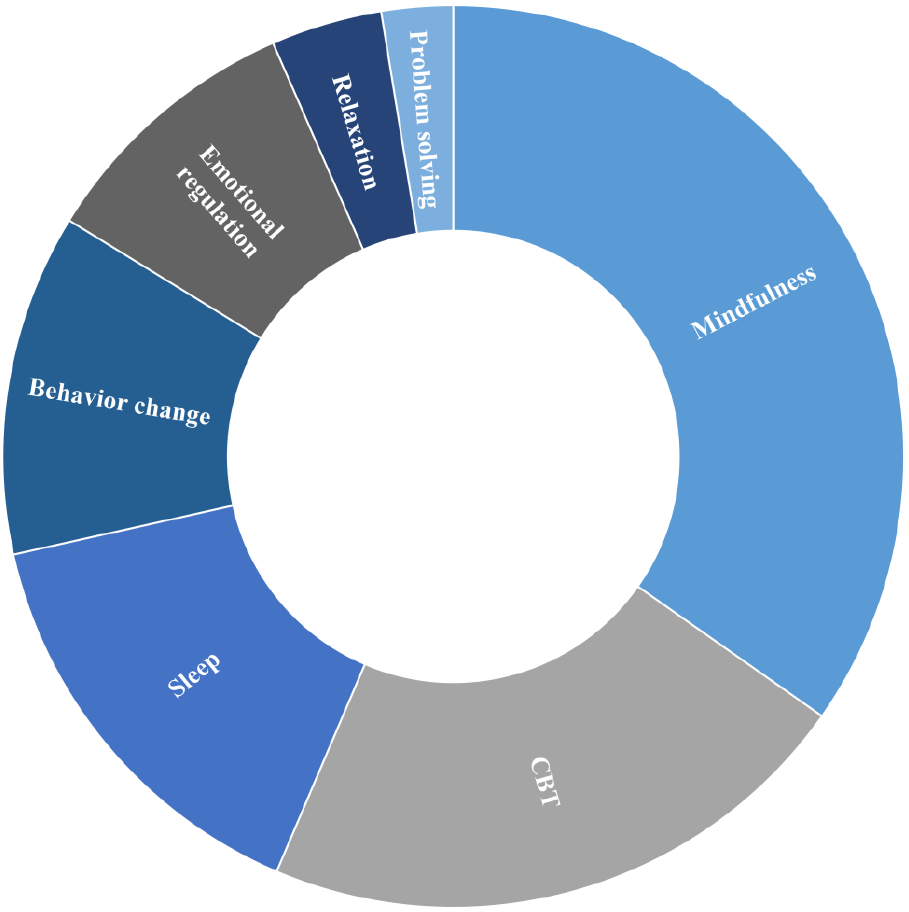

Supplement: Multimedia Appendix 1 [file formative_v6i2e34889_app1.png]
